# De-sync: disruption of synchronization as a key factor in individual and collective creative processes

**DOI:** 10.1186/s12868-024-00874-z

**Published:** 2024-11-06

**Authors:** Julien Laroche, Asaf Bachrach, Lior Noy

**Affiliations:** 1 Center for Translational Neurophysiology of Speech and Communication, Italian Institute of Technology, Ferrara, Italy; 2 Structures Formelles du Langage, UMR 7023, CNRS, Paris, France; 3 Faculty of Business Administration, Ono Academic College, Kiryat Ono, Israel

**Keywords:** Creativity, Disruption, Synchronization, De-synchronization, Collective dynamics, Distributed creativity, Group creativity, Dynamical systems

## Abstract

Creativity is a key skill for the twenty-first century, where the individual and collective imperative to adapt is omnipresent. Yet, it is still unclear how to put creativity theories into practice, which signals a lacuna in our understanding of the pragmatic means by which we get creative. This paper starts from the identification of a number of gaps in the literature. In particular, individual and group creativity are usually treated separately, and the emphasis on the search for novelty seems to overshadow the importance experts give to the disruption of their habitual patterns of behavior. To overcome these gaps, we propose foundations for a unifying framework that takes the perspective of dynamical systems. Specifically, we suggest that de-synchronization, a hallmark of disruption, is an integral part of the creative processes that operate across individual and collective levels of analysis. We show that by conjuring uncertainty, de-synchronized states provide opportunities for creative reorganization. In order to ground this framework, we survey and discuss existing literature, and focus on group improvisation practices (in particular, music and dance improvisation), where partners use the dynamics of their interaction to bring forth a collective performance in real-time. In these practices, disruption by de-synchronization, termed here as ‘problematization of coordination’, is a pragmatic approach used to push the creative process forward. We suggest that this approach might also be relevant in other types of individual and collective creative processes.

“Disturbance is a change in environmental conditions that causes a pronounced change in an ecosystem. [..] Disturbance opens the terrain for transformative encounters, making new landscape assemblages possible. [..] Whether a disturbance is bearable or unbearable is a question worked out through what follows it: the reformation of assemblages.” (from Anne Tsing, “The Mushroom at the End of the World”, pp. 160–161, 2015).

## Main Text

Creativity, the ability to come up with novel and useful products (i.e. ideas, artifacts, or behaviors), is a key skill for the twenty-first century, where the need to adapt to new technologies or new social and environmental conditions is crucial [1]. Creative skills are particularly evident in practices such as improvisation, where the created performance is made up in real-time, often through the interactions between multiple performers who do not know what each other will do next. However, research on creativity usually treats individuals and groups separately. Moreover, the literature associated with group improvisation demonstrates a gap. On the one hand, qualitative analysis and phenomenological investigations reveals that improvisers create by seeking to disrupt their habitual routines and agency [2–5]. On the other hand, the growing empirical literature on joint action often focuses on goal oriented coordination of behaviors between persons [6–10]. In this paper, we aim at bridging the gap between individual and group creativity, between disruption and coordinated activity, and between phenomenological and observational perspectives. To do so, we provide the foundations for a unifying framework inspired by the principles of dynamical systems that bring together literature ‘bits’ that are spread across different domains. In our view, these bits present common features that are operationalized by different methods and discussed with different concepts. Specifically, we suggest that de-synchronization, a hallmark of disruption, is an integral part of the creative processes that operate across individual and collective levels of analysis.

## Introduction

Despite the importance of creativity skills, and the development over the last century of theories of individual [11–13] and group [14–16] creativity, it is still unclear how to put creativity theories into practice. For instance, while creativity is recognized as one of the most important skills in modern education [1, 17], applying creativity research in pedagogical environments has proven difficult [18, 19]. This suggests a gap in our understanding of the *pragmatic* means by which we get creative: what is it that we actually do when we invent novel and useful ideas, artifacts, or behaviors? This gap reflects the fact that creativity research has focused more on the outcomes of creative processes (the creative products) than the dynamics of these processes [20, 21]. Moreover, when a creative process is investigated, it is often portrayed as a *mental quest for novelty performed by an individual* [22, 23]. This description raises three issues that this paper aims to address. First, creative processes are not confined in mental operations: they also involve bodily actions and interactions with the environment [18]. Second, practitioners often develop explicit strategies that aim more at disrupting established patterns, than at searching for novel ones [2–5]. Third, in many cases, it is a group of individuals which creates [24–26]—for instance during brain-storming [27], joint problem-solving [28], innovative team work [29], or collective improvisation practices [30]—and in such cases creativity stems less from individual thoughts or actions than from the *interactions* between individuals [16, 31]. Yet, embodied and distributed processes of collective creativity have been seldom studied [22], let alone those related to the disruption of established patterns [5].

In this context, the International Workshop on The Neural and Social Bases of Creative Movement (IWNSBCM, https://yourbrainanddance.egr.uh.edu/about), where works published in this special issue were initially presented, was welcome. It brought together practitioners and scientific experts that are interested not only in the neurocognitive underpinnings of creative processes, but also in the bodily and the interpersonal processes that many creative activities involve. In many domains, bodily movements and the traces they leave directly constitute the created product, for instance in drawing [32], craftsmanship [33], or sport [34]. For this reason, body-based activities provide a particularly fruitful angle on creativity: movement externalizes an important part of the creative process and brings it to the observable domain, since motion can be captured, measured, and analyzed. At the IWNSBCM, two popular art forms were at the center of shared interests: music and dance. Music and dance are often performed collectively and take the interactivity between movements and the perceptual traces they leave as a source of creation. In particular, improvisation, the real-time creation of an unscripted performance, is transversal to both fields and lends itself well to collective forms. As such, group improvisation is particularly relevant to address the issues raised here. In group improvisation the created product (the improvised performance) is collectively elaborated: it emerges from the interaction between partners. In addition, because the created product strictly coincides in time with the bodily actions that perform it, improvisation enables the simultaneous study of creative products and processes. Finally, the techniques that expert improvisers explicitly exercise and deploy during their performances can strongly inform scientists about the pragmatic means that we can use to get creative together. In short, group improvisation offers a unique window onto the creation of collective products.

The three authors of this paper are cognitive and motor scientists that also have a practical expertise in improvisation (in music, dance and theater respectively), and circulate between the domains of research and practice. In that spirit, the first two authors shared the animation of a symposium at the IWNSBCM with other movement experts (dancers and choreographers) and researchers (philosopher, anthropologist, neuro-linguist, and psychologist). The symposium encouraged mutually beneficial exchanges between practical expertise and scientific knowledge, and aimed at refreshing the perspective that scientific methods hold on dance and dancers.

In the present paper, our aim is to identify and discuss the relationship between desynchronization and creative processes—a question that was raised during the symposium, through the dialogue between practitioners and scientists -. In effect, this relationship has been mostly overlooked in creativity research. More specifically, we suggest that while creativity research has mainly focused on the production of novel and valuable things, it has neglected the role of disrupting established patterns as a critical step in this process. In this context, we identify *de-synchronization*—the loss of the temporal locking whereby a number of elements form a pattern together—as a hallmark of disruption that is an integral part of the creative process. We propose that de-synchronized states, by conjuring uncertainty, provide opportunities of reorganization into novel and useful patterns.

To articulate our proposition within a coherent theoretical framework, we take the perspective of dynamical systems, which that stems from a long tradition in statistical physics [35, 36]. Dynamical systems principles have previously provided useful modeling tools in cognitive sciences [37–39], in particular regarding mechanisms of synchronization [37]. Building upon this theoretical framework we describe how de-synchronization in creative processes scales across individual and group levels of organization [40]. Here, we review and interpret results collected in creative tasks with emphasis on improvisation, reflecting the main interests expressed at the IWNSBCM and our own contributions there. Music improvisation suits the topic of de-synchronization particularly well and will receive a stronger attention, while dance/movement improvisation will allow us to confirm that the observed mechanisms and formulated hypotheses extend across domains of practice. We also build on the pragmatic experience of experts in these fields as a unique window that allows us to address the three issues in creativity research we identified above: the creative role of bodily activity, of interactional processes, and of the disruption of established patterns.

In the following parts, we first provide a quick overview of the literature that points at the general role of disruption of established patterns in favoring individual creative dynamics. Next, we introduce dynamical systems principles that account for the phenomenon of (de-)synchronization, followed by examples of how de-synchronization, by conjuring uncertainty, can help an individual to break away from established patterns and explore, discover and ultimately learn novel ones. We then discuss the literature showing how interpersonal de-synchronization can, by disrupting collective dynamics, expand opportunities of reorganization into novel functional patterns of interaction at the group level. We end this paper by discussing the need to better understand the factors that best allow creative reorganization to emerge through disruption and speculate about the nature of these factors.

## Disruption and creative re-organization of individual dynamics

### Disruption is a core process in creative dynamics

To develop new skills, we often need to inhibit more salient paths that lead to familiar, yet less adaptive actions [41]. Remaining within the zone of what we already know can prevent us from stepping out from our established patterns: we often get fixated on a specific array of possibilities provided by our dominant behavioral and cognitive tendencies, which hinders the potential discovery of novelties [42]. Creative exploration thus requires the inhibition of dominant and non-creative paths [43]. This can be observed in real-time when tracking the search process in a creative foraging game where tiles are rearranged to assemble shapes: players alternate between phases of exploitation where they focus on clusters of shapes that belong to a similar category (e.g., planes or letters) and phases of exploration where they search for new categories [44–46]. In these studies, players vary significantly in their tendency to stay longer in a discovered category (see [45], Fig. 6). This variability among participants might reflect different personality tendencies regarding safety and risk taking. A similar finding comes from ethological studies showing different styles of foraging behavior in birds, differences that might have a genetic basis [47]. These different foraging styles have different costs and benefits. For example, leaving a currently safe exploitation region is risky in the short term, since finding a new and better region could fail. Yet, it might increase the chance of successful foraging in the long run, as the current region will most likely be depleted eventually. In creative foraging, staying in the current ‘safe region’ for too long brings risks of boredom [46]. Interestingly, in the creative foraging game, the quicker players quit a category they were exploiting, the quicker they find new ones: the tendency to disrupt ongoing patterns and leave them thus seems related to the probability of finding novel ones, an issue we will get back to in the final section.

One commonly reported strategy for stepping out of an established pattern is the introduction of constraints [48, 49]. Instead of merely limiting the available range of behaviors, constraints reconfigure the horizon of potential opportunities [49]. In other words, precluding certain behaviors by constraining them can promote alternative ones [48]. Since we spontaneously tend to embrace familiar paths, destabilizing established patterns thus seems to be key to the emergence of so-called creative moments [50, 51]. Pattern disruption entails states of uncertainty that, because they enhance the variability of future potential patterns, are increasingly recognized as necessary doorsteps towards novel paths [52–55]. Interestingly, embracing uncertainty is at the very heart of improvisation practices [56]. As a result, simply taking part in verbal or musical improvisations suffices to help break away from established patterns and enhance creativity, for example in terms of increased divergent thinking, in cognitive tasks performed after the improvisation session [57–60]. Processes that apply in improvisation practices might then be involved in creativity in general, and be applied to train and hone creative skills.

In sum, raising uncertainty by disrupting established patterns seems to enable creative shifts. In this paper, we suggest that processes of de-synchronization are a hallmark of disruption that holds the potential to foster creative dynamics. To describe how de-synchronization conjures uncertainty in a way that can favor creative shifts, we now take the perspective of dynamical systems theory.

### Dynamical approaches to (de- and re-) synchronization

Synchronization, understood as the temporal locking of elements that form a pattern together, is a well-researched phenomenon both experimentally [61] and phenomenologically [62]. It is a domain general phenomenon that operates at multiple levels: physical [63], physiological [64], behavioral [37] and social [61]. Research within these fields is mainly concerned with the emergence of stable organization [63], and de-synchronization, understood as the disruption of synchronized patterns, has received less attention. Yet, synchronization and de-synchronization are complementary processes [65, 66], and dynamical systems principles provide useful tools to capture not only how patterns can form and sustain within complex systems, but also how they collapse and change at various scales of observation (e.g., neural, behavioral, interpersonal [65]). De-synchronization, which can be experienced in first-person and experimented on with scientific methods, can be measured with similar tools to those used to capture synchronization. As such, de-synchronization can serve as a useful probe for studying the role of disruption in creative processes, at multiple levels and from both first- and third-person perspectives. In the following part, we discuss how de-synchronization between the components of a complex system raises uncertainty, and how this can reconfigure the patterns under which the system can reorganize itself and accelerate the transition toward these alternative, and potentially novel, paths.

**Fig. 1:**
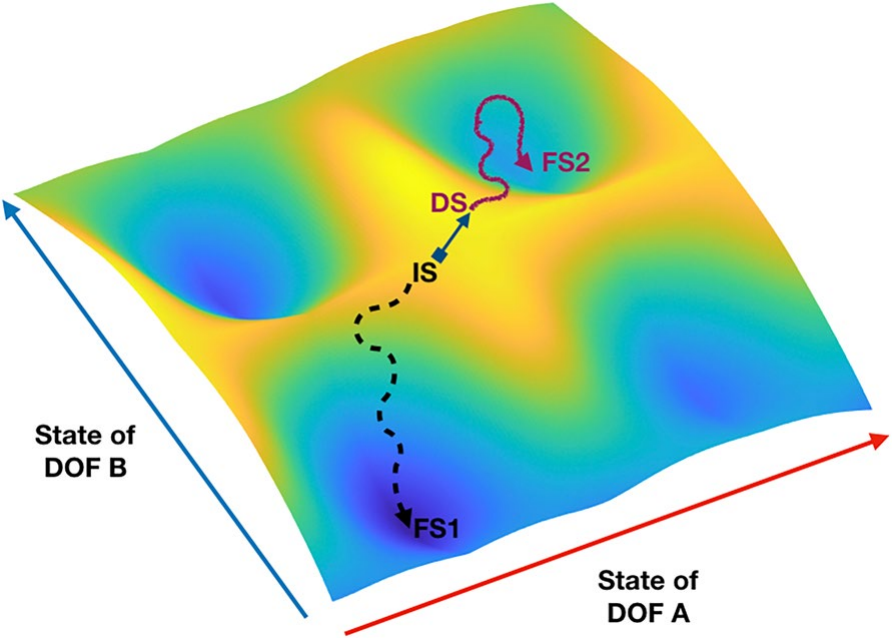
Visual representation of a landscape of attraction given two arbitrary DOFs (**A** and **B**). Dips in cold colors indicate basins of attraction towards which the system is drawn and materialize stable patterns of interaction between the respective states of **A** and **B**. Hills in hot color indicate unstable patterns which the system is pulled away from. When **A** and **B** have certain states, they tend to evolve toward the deep basins the system is close to. The black curved and dashed arrow represents the trajectory of the system given an initial state (IS) and its tendency to follow habitual paths of attraction toward a stable pattern—the final state where it settles (FS1). The curvature illustrates fluctuations that can stem from intrinsic noise present in the system or from other DOF not represented here. In unstable situations, tiny changes in the values of the DOF can nudge the system toward one or the other basins. The violet curved arrow represents the trajectory of the system when it is first disrupted (blue arrow) and put in such an unstable situation, at the crossroad between several potential alternatives (disrupted state, DS), and then nudged toward a pattern that is stable but usually less attractive (the second final state, FS2). The DOF B could be a parameter of the environment (including another person with whom one interacts with), expressing the fact that certain patterns become attractive and stable thanks to the process of interaction with our surroundings and in the context of our own ongoing activity

We can describe a complex system as a network of interdependent parts or “degrees of freedom” (DOF), which designate the subprocesses that parametrize the behavioral evolution of the whole system. Key to the explanation of pattern formation, dissolution and change into new ones are the temporal contingencies amongst these DOF [37, 65]. Specifically, there is a tension between two coexisting tendencies: DOF tend to couple and synchronize their activities on the one hand, and to obey their own intrinsic dynamics, causing de-synchronization, on the other hand [37, 67]. This tension was first observed in the oscillations of fish fins: they obey alternatively to a « magnet effect» where the fins synchronize with each other, and to a « maintenance effect» where each fin moves at its own pace [68]. The tendency of DOF to couple and synchronize their activities at the micro-level allows for the emergence of coherent patterns at the macro-level. Some of these patterns are more stable than others: they assemble more easily, persist for longer times, are more resilient to perturbations, and recur across a variety of situations [69, 70]. The differential stability of potential patterns gives rise to a landscape of attraction in the context of which tendencies arise [65]: the system is pulled toward the basins of attraction formed by stable patterns, and pushed away from regions of instability (see Fig. 1). Repetition and reinforcement can strengthen the shape of this landscape even further [70], accounting for the tendencies of complex living systems to recurrently converge and fixate on habitual patterns. In this context, the search for novel patterns appears to be in tension with the constraints exerted by this dynamical landscape: attracting tendencies hinder the exploration of functional patterns that reside further away from basins of attraction. In short, dynamical systems principles, and the models of habitual behavior they inspired cognitive scientists with [70], help us to better understand the importance of escaping the pull of established patterns in order to discover novel ones.

The complementary tendency of DOF to behave independently, according to their own intrinsic dynamics, provides living systems with the essential ability to flexibly switch between behaviors. In effect, it counteracts the coupling tendencies of DOF by de-synchronizing their activity, which introduces disorder and uncertainty in the pattern they were forming, eventually leading to its dissolution [37]. By compromising the deterministic attraction of previously established patterns in this way, uncertainty allows the system to momentarily escape the influence of its habitual tendencies. This gives the system a chance to change and start over from resetted initial conditions—at least for a transient period of time. Specifically, DOF get a unique opportunity to interact and reorganize in different ways. In effect, once a pattern is dismantled, complex systems operate at the edge between a plurality of potential patterns that exert a weak attraction over their dynamics [65, 67]. In this regime (termed metastability [67]), and because in complex systems multiple parameters conspire together and weigh in the evolution of the system’s behavior, the system’s dynamics become more sensitive to smaller changes that occur either in its internal dynamics or in the environment. Small and momentous fluctuations in one of the parameters that govern its behavior can suffice to nudge the whole system toward alternative paths. As such, DOF re-organization can potentially reveal novel patterns that the saliency of more familiar ones might mask (see Fig. 1, violet curved arrow). In such cases the system’s behavior becomes less predictable and is thus more prone to creative moves in a wider variety of potential directions.

In sum, the de-synchronization of DOF is a key step that can stimulate the exploration of alternative possibilities: it introduces uncertainty that refreshes the system’s perspective on its potential future paths by altering the landscape of attraction that orients its behavior. Specifically, the de-synchronization of DOF transiently relaxes the long-term tendencies that determine the system’s behavior, allowing it to reorganize into potentially novel patterns that established ones might have previously masked.

### From de-synchronization to creative re-organization of agent-environment coordination patterns

For simplification, we described complex systems from the point of view of their internal processes. However, as hinted above, complex systems such as neurobiological agents are not closed systems: they operate and maintain their existence thanks to the couplings they establish with their environment [71]. DOF that participate in behavioral patterns are therefore situated beyond the boundaries of the skull and flesh of an agent. To illustrate how de-synchronization processes and uncertainty in the coupling with the environment help weakening established patterns and opening up an array of novel opportunities, we now look at two empirical examples where signatures of uncertainty and de-synchronization with the environment precede creative shifts.

First, problem-solving tasks can provide interesting cases where the discovery of a novel pattern follows the dissolution of a previously established one. For instance, when participants observe a chain of interlocked gears and have to guess in which direction the last one will rotate, they first solve the task by mimicking the motion of each gear with their hands [72, 73]. After a while, they tend to discover a new strategy that leads to a much quicker solution: as each gear moves the next one in the opposite direction, for every second gear in the chain the rotation is identical. Two statistical signatures present in hand kinematics are observed just before this discovery. First, entropy (a measure of uncertainty) increases: this reflects critical instability within the current pattern, giving a chance for change to occur. Second, power-law behavior [74], a statistical index that quantifies the strength of the relationship between the activities that occur at multiple levels of organization within a system, increases. This constitutes a marker signifying that the system is driven more and more by the interactions between its DOF, and therefore prone to explore and susceptible to reorganize into a variety of different possible patterns. Once behavior has transited toward the new hand motion strategy, both statistical indices quickly drop: reorganization into a new functional pattern has dissipated the exceeding uncertainty. Critically, enhancing uncertainty by making the gear system jitter accelerates the discovery of the new solution. In other words, pushing the sensorimotor coupling between the agent and the gears toward de-synchronization helps to shift away from a previously established pattern and accelerate the discovery of a new one. For that to happen, the previously established pattern had to be dissolved, leaving enough room for a transition toward a re-organized movement pattern. However, the discovered pattern does not randomly arise from nowhere. Instead, not only does the pattern belong to the motor repertoire of the participants (on a latent form at least), but it was also already implicitly present, or embedded in the previous pattern of interaction with the task environment (i.e., the pattern of alternation of the direction of the successive rotations). In other words, the creative re-organization can emerge because there is a resonance between the agent's sensorimotor skills, the structure of her task environment, and the properties of her ongoing activity.

A second empirical example, how boxers hit heavy bags, analyzed by [75], illustrates how uncertainty in the coupling of the agent and the environment can open up a field of novel opportunities. At certain distances from the bag, only a limited range of hits can be performed, increasing the predictability of the boxer’s moves. However, at a critical distance, the coupling dynamics between boxer and bag become less determined and a wider set of opportunities becomes available, decreasing the predictability of the boxer’s moves. Moreover, when the bag was stochastically moved by an assistant, novel action possibilities were discovered at other distances too [34]. Just like in the gear system experiment, introducing uncertainty in the coupling between agent and environment by noisy fluctuations was sufficient to expand the range of action opportunities. Tiny details can thus pull the sensorimotor coupling of the boxer in various directions, making novel combinations of actions available for discovery and learning. These combinations are particularly functional as they can hardly be predicted by an opponent, reflecting the direct usefulness (decreasing the ability of the opponent to avoid the hit) of this creative discovery. We note that the emergence of novel patterns, despite requiring to transiently escape the influence of established ones, still relies on the repertoire of actions an agent has at her disposal, and on the possibility of combining these actions together. Paradoxically, it takes a whole array of habitual skills, and the ability to combine them together, to creatively defeat the habitual tendencies which engulf our behavior.

Generalizing from these two examples, it seems that becoming more creative might require learning to exploit the dynamics of our behavior and its coupling with the environment to transiently strive toward more uncertainty. This uncertainty helps weaken or dissolve established patterns, escape their attraction and afford the opportunity to recognize novel ones within reach. To discuss how disruption can instill this kind of uncertainty in improvisation, we now discuss the literature on two improvised practices (music and dance) at the individual level.

### Disruption and uncertainty in improvisation

Studies of music improvisation have revealed two, seemingly contradictory, findings. On the one hand, work in cognitive modeling [76, 77], systematic musicology [78] and neurosciences [79, 80], have demonstrated that improvisers rely on a pool of pre-learned sequences that they integrate rather automatically in the course of their performance, giving a sensation of « effortless mastery» [81]. On the other hand, first-person reports of experts show that improvisers seek to escape their habitual routines by disrupting their own agency: they release part of their control and conjure new constraints with which they can cope creatively [2, 3, 82].

From a dynamical systems perspectives, we can consider control and spontaneity [83] as well as stability and flexibility as complementary poles rather than contradictory ones [65]. Dynamical systems principles have gained popularity in accounting for perceptuo-motor mechanisms associated with music [84], and Borgo has pioneered the idea of interpreting improvised performances in terms of patterns living on the edge between stability and instability [85, 86]. Relying on his own experience as an improviser [87] and on the experience of others through field work [88], Borgo has published theoretical [89] as well as empirical works that blend qualitative music analyses and quantitative analyses of audio signals using concepts and tools from non-linear dynamical systems principles [85, 90]. He has notably insisted on the necessity to surf “on the edge of chaos”, between the stability that past experiences and current interactions with others help to foster, and the instability instilled by surprising fluctuations. Specifically, Borgo insisted on the importance of weakening the attraction of certain patterns in order to break them and engage with others [85]. Along these lines, quantitative analyses of the melodic content of improvised sequences confirmed that patterns that were judged more creative had a higher degree of (melodic) uncertainty [91]. Uncertainty and the disruption of pattern stability have thus been progressively considered to be core catalysts of musical creativity [92, 93].

As such, improvisers, and the couple they form with their instrument, can be seen as self-organized systems that reorganize themselves to cope with the evolving constraints they face [94, 95]. Improvisers find creative paths by skilfully navigating the affordances that their own dynamics and their coupling with the environment puts at their disposal [96]. First-person perspective interviews corroborate this view: improvisers explicitly report that they find creative situations of uncertainty by inducing or exploiting instabilities in their interaction with their instrument and the sonic outputs it affords [2, 3, 82, 97, 98]. Experiments have shown that inducing nonlinear relations between the musician and its instrument helps bring forth surprising interactions that foster creative behaviors [98]. Overall, this literature suggests that music improvisation relies less on the optimization of sensorimotor control over the instrument but rather on the flexible modulation of that control. This allows performers to surf on the edge between pattern stability and the creative potential of unstable situations in which they need to reclaim their agency in a creative manner [2, 3].

In dance improvisation, performers directly use their body movement to interact with the material space and other dancers, without the mediation of a sound producing instrument. As dynamical systems principles have mostly been applied to human behavior through the modeling of motor control [99, 100], such a framework is particularly relevant here. So far, research on dance has seldomly integrated dynamical systems principles, but a few authors have shown how a better understanding of dance learning and motor creativity could benefit from seeing the performer and its relation with the environment as a self-organized system [101–105]. Other studies have focused on the effect expertise has on body movements coordination, particularly in relation with auditory streams [106–109]. For example, Miura and colleagues point out that novices tend to enact stereotyped, non-original movements [105]. They explain that as a tendency to spontaneously entrain to synchronized patterns of motion, from which novices have difficulty freeing themselves. Practicing dance, on the other hand, would help to escape unintentional formation of specific patterns, thereby allowing dancers to overcome pre-existing pattern tendencies [105]. For instance, while expert street dancers show greater sensorimotor coordination abilities compared to novices [106–109], this seems to be made possible by their ability to lower muscle co-contraction and introduce phase lag between moving joints, as if they were avoiding a kind of self-entrainment by de-synchronizing with themselves [105, 109]. In that sense, dance practice is not so much about stabilizing specific patterns than it is about learning to flexibly rearrange possible patterns of motion, and free the self from its own intrinsic dynamical constraints [110]. In that regard, movement improvisation is portrayed as an intervention method that can foster the actuation of creative potentials by overcoming “states of inertia” [111].

In the context of dance improvisation, dynamical systems principles have been employed to account not only for the sensorimotor constraints that exert their influence over dance performances, but also for the cognitive constraints that implicitly weigh on the performers [112–114]. Hansen points out that “performance generative systems”, where simple systems of rules are set to shape improvisation, are highly demanding in terms of executive functions [114]. Such constraints can attract the formation of certain patterns more than others, thereby limiting the creative potential of performers. However, as constraints imposed on a performer bias the self-organized dynamics of their performance [113], imposing extrinsic constraints can help gain awareness of the influence of intrinsic cognitive ones, which can then be creatively manipulated or simply inhibited [113]. Extrinsic constraints might thus preclude the habitual paths intrinsic constraints lead to, thereby helping to widen the range of movement possibilities during body expression [115]. Dancers can be said to directly play with the constraints that influence their coordinated patterns of motion (with the self, the environment, and as we shall see later, with others), and disrupting familiar patterns should be an efficient way to promote novel ones [111]. Indeed, dance improvisers, just like music improvisers, report that to seek novel patterns, they alter their own agency. Facing uncertainty and new demands motivates them to reclaim control in a creative adapted manner, and that they do so by delegating part of their control to their environment [2, 3, 101].

In sum, improvisers—musicians and dancers alike—appreciate, seek for, and voluntarily solicit situations of uncertainty in which their habitual agency is disrupted: this forces them to reclaim agency [4] and reorganize their sensorimotor performance in a creative manner [2, 3], thereby endowing disruption with a creative potential. However, the practice of improvisation is deeply linked with group interactions. Dancers and musicians alike report that, even when they perform in solo, they often emulate an otherness so as to destabilize and step out from their own established patterns [3]—as if they were de-synchronizing with the self. In group improvisation, and in group creativity in general, others could therefore be the destabilizing source of DOF that instills and fuels a performance with the kind of uncertainty that motivates creative reorganization. Next, we discuss how the dynamics of interaction between persons, in particular the de-synchronization of their activities, can elicit uncertainty, and thereby disclose novel possibilities.

## The creative group: disruption of collective dynamics and collective disruption of individual dynamics

### The group as a complex and creative system

A strong advantage of dynamical systems principles is that they account for mechanisms of interaction across multiple levels of organization [116], whether the interacting components are groups of neurons, limbs of the same body, a person and her environment, or multiple persons. As we discussed above, at the individual level, the landscape of attraction which underlie our behavior is parametrized by properties of the environment—for instance, the distance of the heavy bag from the boxer, or the tempo of a metronome one has to tap into [65, 75]. In the case of joint action, where two or more individuals collectively pursue a shared goal, each person is part of the environment of the other, and partners can be thought as DOF that form a complex system together. Such interactions give rise to patterns of collective dynamics [117, 118] which shape the behaviors of the group members in return [119–121]. As such, reciprocal interaction alters the landscape of attraction that underlies each of the interacting persons’ behavior [122]: when we interact with others, we are inclined to behave differently than when we are alone. If interacting with others does modulate the differential attractiveness of potential behavioral patterns, then interacting might also disclose patterns of behavior that lie further away from our habitual paths. This has been observed using minimalistic artificial agents that freely explore their environment: interactions between agents increased the diversity of the behavioral regions that they visited [123]. Similarly, a weak nonlinear coupling between a human and a virtual partner suffices to help the human partner to perform rhythmic patterns that did not belong to their initial repertoire [124]. Modeling behavioral change through the lens of dynamical systems principles has been fruitful in clinical practices as well: we can understand the role of the therapist as helping the client to escape the attraction of some habitual patterns of behavior and to shift to new, healthier ones [125]. In the next paragraph, we discuss the literature showing how the dynamics of interaction can enhance creativity at the level of the group itself.

In groups, individual partners and their intrinsic processes constitute the DOF whose interactions give rise to dynamical landscapes at the collective level. Consequently, certain patterns of interaction are more stable than others, and are more prone to be adopted by coupled partners when they interact together [122]. Here too, the spontaneous tendency to couple with others (the ‘magnet’ effect) can lead a group to spontaneously adhere to established patterns, which can potentially hinder the capacity of that group to get creative by exploring alternative paths. Yet, this tendency to couple with others is complemented by the tendency to obey one’s own dynamics (the ‘maintenance’ effect discussed earlier [68]). As agent-based simulation and recent theories suggest, the heterogeneity of individual dynamics within a group loosens interpersonal coupling in a way that should favor creative group reorganization [126, 127]. In effect, diversity, for instance in terms of gender [128] or task experience [129], have been shown to benefit collective creativity tasks, and so does minority dissent in groups where members are highly engaged in their interactions [130, 131]. The latter observations show that creating collaboratively is not so much about coordinating our actions or thoughts, but depends more fundamentally on the quality of our participation in the regulation of not-so coordinated interactions. While some have focused on the tracking of interpersonal synchronization during problem-solving [132] and creative tasks [133], communication breakdowns and the subsequent attempts to repair them might actually foster meaningful collective creativity even more [134]. In sensorimotor experiments it has been observed that in groups with more heterogeneity, individuals coordinate their action less strongly. It is therefore possible that the heterogeneity of individual dynamics in group creativity activities favors interpersonal de-synchronization, thereby inciting reorganization into novel, potentially fruitful patterns of interaction. Supporting this interpretation, it has been shown that dyads can perform better at creative tasks when the partners’ body movements are more loosely coupled [135], whereas synchronization of motor activity reduces dissent and creativity in subsequent tasks [136].

In sum, group interactions can disrupt the dynamics of both individual and collective behaviors in a way that fosters their creativity. Group improvisation provides a particularly intriguing context to observe such phenomena: partners couple their behaviors across time to invent, together and on the spot, a new performance that is based on the patterns of their interaction [30]. This setting makes the contingencies between partners’ behaviors particularly crucial and central. Not only does it allow for genuinely collective patterns to emerge, but it also amplifies the magnitude with which the dynamics of interaction can shape individual behaviors (compared, for instance, to imitative joint-music making [137]). Moreover, the need to coordinate actions in time can make moments of de-synchronization particularly evident during improvisation. Below, we discuss the creative role of disruption in group improvisation by having in mind two distinct scales at which creative shifts can be observed: (1) at the individual level, where the dynamics of our own behavior can shift under the influence of our interactions with others and put us on a path toward patterns that are novel to us personally (2) at the group level, where collective patterns of interaction can shift toward forms that are novel to the group. To articulate this discussion in the next paragraphs, we start by recalling the importance of interpersonal coordination in musical ensembles, and how this suits modeling through dynamical systems principles. Next, we discuss the importance of disruption and more particularly de-synchronization in fostering the creativity of individual performers. Then, we focus on creativity at the group level to further discuss the articulation between de-synchronization and creativity. Finally, we perform a shorter but similar survey of literature in the domain of dance to show that observations recur across two different disciplines.

### Improvisation as a coordination problem

The fact that a group of improvisers embarks on a collective performance without knowing in advance what each other will do places a burden on the temporal alignment between their respective behaviors. As such, group improvisation is sometimes seen as a “coordination problem”: improvisers need to interact first and foremost to make their intentions and their sonic gestures converge toward stable, cohesive forms, or “collective sequences” [138, 139]. Researchers have accordingly sought to quantify interpersonal coordination between improvisers by looking at their sonic outputs [140–143], their body movements [144–146] and other physiological processes [147]. Synchronization between performers' body movements, for instance, is a strongly structuring factor: it reflects aspects of the musical structure [148] and the interacting roles partners endorse (e.g., leading or following; [149, 150]). Interestingly, synchronization between bodies can even be stronger or follow more regular patterns in free forms of improvisation compared to pulse-driven ones [146, 151]. This increased interpersonal synchronization is noticed by naïve external observers [152], and influences their judgment of a performance [153]. Increased interpersonal synchronization also influences performance judgements by expert observers [146]. These results support a dynamical perspective on improvisation. According to this perspective, collective patterns are distributed among performers and emerge from the self-organization of their mutual interactions [94, 145, 154]. In a similar vein, jazz improvisation duets and their audience enjoy the performance more when musicians interact bidirectionally, rather than unidirectionally (with one musician playing with a recording of the other) [155, 156]. The enhanced quality of an interactive performance was also demonstrated using agent-based models showing how improvised mutual interaction between agents can lead to the formation of complex tonal patterns [157].

Given the importance of interpersonal synchronization in musical ensembles, models of music coordination between multiple agents inspired by dynamical systems principles tend to see the disruption of synchrony as a factor that hinders performance [158]. In this view, disruption is ‘risky’ as it might lead to mis-communication or diverging representations. However, as stressed in the current paper, synchronization can also impede creativity, in particular during improvisation. In effect, interpersonal coordination of behavior is often not a goal or a challenge that needs to be purposefully attained: when studied in controlled sensorimotor experiments for instance, it occurs spontaneously, without any intention to do so [117] or even despite contrary intentions [159]. As such, the spontaneity of interpersonal synchronization might place the group in situations where it is attracted toward stable, established patterns of interaction, or incite performers to stay within the comfort zone of their own established individual tendencies, preventing the emergence of genuinely novel patterns [5]. In fact, even in a minimal environment of rhythmic dyadic improvisation, certain preferred patterns quickly emerge [160]. As such, synchronization could therefore become a problem by exerting an attraction that hinders the emergence of more original patterns. From this perspective, improvisation could be seen as a coordination problem where synchronization must both be achieved and later be defeated to allow the emergence of original patterns. Even if a strong sense of coordination is necessary to carry an improvised performance from its beginning to its end, genuine creative shifts might then reside in different mechanisms that operate transiently. This corresponds to the “articulation challenge” described by Cannone and Garnier [139, 161], where performers have to find and switch to different patterns together.

There is empirical evidence in support of this suggestion. It was observed that despite the strong importance of interpersonal synchronization during collective improvisation, performers often loosen their synchrony, or play with their a-synchrony to shape the temporal dynamics of their collective performance [162–166]. In a multi-level study, both the quantitative analysis of tempo coordination between a drummer and a bass player and a transcript analysis of their interview during the studio recording of an improvised track showed that their preferred relational timing was *not* the synchronous state. Instead, these musicians co-negotiate the temporal contour of their performance by oscillating around each other, pushing and pulling the thread of time they weave together [141]. The playful coordination of fluctuations and asynchronies might thus be the perceivable fingerprint of musical interactivity [141, 167]. More than a sensorimotor approximation, subtle fluctuations of synchrony at short timescales might in fact open a space for the exploration of novel patterns of expression [168].

To summarize, viewing collective improvisation solely as a coordination problem (or a synchronization challenge) might place too strong a burden on prediction mechanisms [169]. Such an emphasis on prediction fails at catching the essence of creativity during improvisation, where surprise is sought for rather than avoided [169]. To further discuss the role of de-synchronized states in musical improvisation, we first take a look at how such states can motivate and foster creative reorganization in individual members of a group of improvisation. To do so, we take the example of novice improvisers, who have a smaller repertoire of techniques to step out of their established paths, and their interaction with an expert, who has developed skills in igniting pattern shifts.

### Improvisation as the problematization of coordination

While experts’ reports provide strong insights into the processes they deploy during creative activities, novices offer a different and valuable angle. How can they create with a more limited repertoire of techniques and motor sequences? In this part, we discuss how novices’ creativity is hindered not so much by their lack of “vocabulary”, but by the high amount of determinism that characterizes their landscape of attraction. In addition, we show how their repertoire can be enriched, and their improvisatory behavior enhanced, not by accumulating discretely learnt sequences, but by altering their landscape of attraction and making new attractors emerge from the disruption of the dynamics of their interaction with others. We demonstrate this idea using a case-study, the Kaddouch pedagogy, where improvised interactions between a teacher and a learner serve as a method for creative learning [170–172].

Qualitative analyses of dyadic improvisation between a teacher and young learners in the context of the Kaddouch music school show that children quickly manifest idiosyncratic tendencies [5, 173]: they are prone to embrace the most habitual and spontaneous paths that their sensorimotor repertoire offers. On one hand, the ease with which they can enact these patterns allows them to easily participate in collaborative creations such as group improvisation. On the other hand, only a few stable patterns shape the landscape of attraction of their behavior. The salience of these patterns tends to determine novices’ behavior to a large extent. As a result, their intrinsic dynamics limits the flexibility of their behavior, hindering the exploration of more creative paths [5, 174]. To help students learn to create with others, pedagogues of the Kaddouch school improvise with the learner and use the impact of the interaction on the learner to guide their behavior. Teachers first synchronize with the learner, and this joint pattern offers a supporting scaffold on which learners can further stabilize their behavior. In other words, learners can rely on the DOF provided by the teacher to ease the coordination of the pattern they play, allowing them to express themselves with more freedom and spontaneity. This synchronization phase is also an important part of the creative process from a socio-affective point of view: it allows the teacher to enhance the learner’s confidence and trust in the creative interaction. Learners, however, often have difficulties stepping out of the pattern they enact during this phase, and tend to show signs of boredom and frustration in such situations. To prevent learners from being trapped into their stereotypical habits, the pedagogue jeopardizes their synchronization by shifting to a musically different pattern that makes the learner’s pattern ‘out of tune’ or rhythmically wrong. Disrupting interpersonal dynamics destabilizes the learners’ own behavioral dynamics: the previously stable, attracting pattern becomes difficult to maintain, unstable in that new context. The reconfiguration of the constraints that are exerted over the student’s dynamics makes room for a more diverse horizon of behavioral opportunities. Post disruption, alternative patterns become easier to access as they are now less shadowed by the attraction of the stronger habitual tendencies. In other words, the uncertainty conjured by the process of de-synchronization emancipates the learner from the salience of their most established tendencies. As a result, and as demonstrated with qualitative analysis of these interactions, moments of de-synchronization and their interactive co-regulation are conducive to creative shifts where students suddenly discover and display behavioral patterns that did not belong to their repertoire before [173]. In some cases, the modification brought by the teacher does not suffice and the learner keeps falling back into the attraction of the most salient behavioral patterns [5]. The new constraints imposed by the teacher must be sufficiently strong and should be maintained so as to keep the learner away from her most stable tendencies until an alternative solution has been enacted. Moreover, the disruption should help reveal features that are resonant enough with the existing repertoire of actions of the learner so that a behavioral solution can emerge and be enacted with enough stability to keep the improvisation going. In fact, even when learners suddenly enact patterns that seem genuinely novel to them, they still borrow noticeable aspects (e.g., tonality or patterns of note groupings) from their spontaneous tendencies [173]—as if similar DOF had been recruited but their relationship had been reorganized in a new manner. As such, the disruption caused by de-synchronization must not entirely hinder the sensorimotor agency of the learners. On the contrary, the disruption must help them navigate their own potential by unveiling certain affordances that learners can grab on to, and use to scaffold novel patterns. The DOF that the teacher provides to the interaction must thus keep constituting a support on which the learner can rest. Under such circumstances, disrupting synchronization at the interpersonal level becomes an efficient technique to incite behavioral reorganization at the individual level, which helps enrich individuals’ behavioral repertoire by motivating the exploration of alternative, potentially novel paths [5]. In that sense, the mechanisms of interpersonal entrainment can be said to foster adaptation and exploration [175] but in a dialectical fashion: interpersonal dynamics of coordination can be exploited to destabilize another person’s own dynamics, and to re-synchronize the dyad or group differently by meeting new coordinative constraints. More than a coordination problem, the creative challenge imposed by group improvisation can be described as the problematization of coordination. Whereas creativity can be sometimes seen as a problem-finding skill more than a problem-solving one [176], we propose to go one step further and to consider creative skills as a problem-making activity: the intentional troubling of our own (or others’) habitual, established paths.

In this part, we have discussed how de-synchronization of interpersonal dynamics can foster the creativity of an individual. However, in group improvisation, the object of creation is collective by nature: it is not an individual quest for novelty. Below, we discuss how de-synchronization between the members of a group can help them become more creative together.

### Creative group dynamics in music improvisation

The above observation in the context of pedagogy with novices and their interpretation in dynamical terms resonate well with the pioneering work of Borgo and his view of expert improvisation as the navigation of potential dynamics on the edge of breakup [85]. Canonne and Garnier [138, 139] also proposed a model for collective free improvisation inspired by dynamical systems principles: collective behavior is described as alternating between collective sequences, evoked above (see subsection ‘Improvisation as a coordination problem’) and assimilated to an attractor in the landscape of attraction of the system, and phases of discoordination that reflect the absence of such an attractor. According to Canonne and Garnier, in groups of expert improvisers, stable collective sequences tend to end either because coordination is too fragile to be sustained (the coordination collapses unintendedly), or because of lassitude (the group or some members intend to change patterns) (see also [5]). Such phases of de-synchronization seem like an ideal place for transition to occur. To conjure such de-synchronized phases, improvisers use intentional strategies such as what the authors name “densification”, which consists in introducing more complexity to the ongoing sequence to motivate a transition [139]. In the light of the previous elements of this discussion, we interpret this increase in complexity as a strategy to increase uncertainty and destabilize synchronization, so as to offer novel opportunities of reorganization between the DOF that are distributed across the group members.

The model of Cannone and Garnier [138, 139] agrees with the observation made above in pedagogical contexts: the teacher helps the learner to navigate the potentialities of their own landscape of attraction by using the dialectic between techniques of de-synchronizing and re-synchronization. In addition, because both players have to co-adapt, and navigate through their own intrinsic dynamics to regulate their collective dynamics, they can be each affected by the disruption in a creative manner. As a result, not only does the teacher acknowledge being affected and inspired by the disruption she introduces and the feedback she gets from the learner, but this process also encourages the discovery of novel patterns at the dyadic level: patterns that none of the individual partners expected emerge from the dynamics of their interaction [5, 174].

In the case of “densification” and in the case of the disruptive techniques used by a teacher, improvisers have a strategic intention to de-synchronize. In other cases, de-synchronization emerges from the misalignment of intentions. In effect, partners in an improvisation often hold different perspectives on their shared performance [141, 177, 178]. In the course of improvisation performed by large ensembles of musicians, partners might have diverging intentions regarding their will to support the stable coordination of their collective pattern of behavior. Interestingly, it is in those moments when intentions are the most de-synchronized that the group tends to shift toward novel patterns [179]. By constraining performers to shift their respective pattern, dissensus [180] might be a key in introducing the kind of uncertainty whose shared experience motivates creative group reorganization [181]. Instead of a mere challenge to coordination, we see mis-communication and the mis-alignement of intentions and behaviors as opportunities to repair coordination [134] in a different, potentially better, and more original way.

In short, during music improvisation, the dynamics of interaction and their disruption through de-synchronization provide a scaffold for discovering novel sensorimotor patterns as a group. The observations and interpretations formulated above in music improvisation reflect well those raised in the more general field of cognitive creativity activities (see subsection “*The group as a complex creative system*”). To further highlight how such mechanisms function across domains, we now turn to dance and movement improvisation.

### Creative group dynamics in dance improvisation

Dancing is often performed as a group, and is often coupled to music. For this reason, research tends to study the synchronization abilities of dancers [106–109]. Interestingly, even in extreme forms of synchronized dance such as artistic swimming, feelings of togetherness are fluctuating in a way that motivates the constant co-adaptation and restabilization of the transiently disrupted performances [182]. On the other hand, studies using rhythmic dance [30] or free movement improvisation [159] have shown how difficult it is to escape the entrainment to each other's movements. Moreover, simply precluding the visual feedback of one’s own movement to focus the attention of partners to their interaction suffices to increase interpersonal synchronization of freely improvised movements [183]. Group dance improvisation exemplifies these intermittent dynamics. When improvising movements together, through mutual influences, we share agency [30, 184] and make meaningful forms of coordination emerge at the collective level [185]. Dance improvisers might thus get carried by the collective dynamics of their interaction and experience a form of group flow where intentions get distributed across partners and during which their shared creativity gets enhanced [184, 186, 187]. However, such moments of flow are intermittent, if not rare [187], and improvisers deploy diverse cognitive and embodied strategies to cope with the communicative demands of the situation [188].

One of the ways through which dance improvisers communicate creatively is by shaping the temporality of their performance together [189]. Like musicians, dance improvisers can actively and playfully alternate between synchrony and asynchrony to form coordinated patterns of interaction [102]. This dialectical relation between stability and flexibility, as well as the spontaneous formation of patterns of interaction in general and interpersonal entrainment in particular, naturally call for interpretation through the lens of dynamical systems principles, where the dancing group is seen as a system that constantly re-organizes itself in the face of constraints [103, 112, 190]. Experiments have shown that shifts between tendencies to explore and to exploit patterns could emerge from the dynamics of interaction when imposing simple rules on partners [191]. Most studies have used contact improvisation (CI) as a testbed, a practice of free collective improvisation where partners in physical contact explore spatio-temporal paths of uncertainty by letting the dance evolve via weight shifts and wilful letting go of individual stability. The non-linearities of the dynamics of interaction within and between dance partners are thus thought to explain both the self-organization of movements patterns and the transition between them at the collective level they form [102]. Specifically, the collective system partners form tends to be attracted toward patterns that depend on their individual and relational characteristics, while the reorganization of the DOF involved allows for the exploration of novel emergent paths [103]. Abrupt, creative reorganization can be ignited by constraints, but they can also limit the emergence of novel moves when they are too rigid, as revealed by dynamical analysis of the series of body configurations used across a performance [192, 193]. One creative source of disrupting constraints is the partner herself. In one study, dancers were first asked to improvise in solo, and dynamical analysis of their kinematics revealed many repeated patterns, reflecting personal attraction toward certain movements. However, when they practiced contact improvisation (in duo), their behaviors switched to a more exploratory regime where no repetition of the movement sequences were detected. Interaction with another seems to enable the bypassing of personal tendencies and thereby strongly stimulate (motor) creativity [194], as the dynamics of interaction spontaneously move us across our dynamical landscape of attraction. According to the authors of that study, and in line with phenomenological work presented earlier [2, 3, 101], letting oneself be influenced by the other might be a mark of expertise in such creative performances. By blending subjective reports of expert dance improvisers in CI with biomechanical analyses of their performances in quasi-experimental situations, Kimmel and colleagues have also studied the complex dynamics at work in CI [56, 195, 196]. Their research shows that dance improvisers, just like musicians, solicit situations where their coupling dynamics is uncertain and allows them to go beyond their familiar paths or the spontaneous tendencies that attract their collective patterns. More precisely, they tend to destabilize ongoing patterns of collective dynamics so as to solicit surprising ones and renew their interaction patterns constantly [56]. What the first-person reports also revealed is that dancers have at least an implicit understanding of these dynamic principles and that they play with these properties to enter metastable regimes and pursue their interactions creatively.

Nonetheless, the study of dance improvisation is still in its infancy and faces the challenge of ecological complexity. The Mirror Game (MG) paradigm has been increasingly employed to address this challenge and quantitatively study movement improvisation. Originally proposed by Noy and colleagues [197, 198], it allows researchers to reduce improvisation’s inherent complexity by using a minimal form of creative interactions. In a typical MG protocol, two partners have to imitate each other while improvising movement, most often by displacing their finger or arm on one dimension. This paradigm allows to probe interpersonal coordination strategies in non-verbal creative collaborations and has proved useful in revealing various patterns of movement interaction related to interpersonal roles such as leading, following or simply interacting mutually without decisional hierarchy [197]. MG research has also revealed that improvisation experts distinguish themselves in such a situation from novices regarding the sensorimotor strategies they employ (e.g., smoothing movement trajectories and making them more predictable). This reduced context eases the capture and analysis of movement data, yet still allows enough freedom and complexity to let creative shifts emerge. Kinematic analysis of a 4-persons version has, for instance, revealed the emergence of “collective sequences” [189] similar to those observed in more ecological group music improvisation [139, 199]. However, even simple dyadic interaction makes it possible to identify stable and idiosyncratic motor signatures that spontaneously structure and attract each person’s movement dynamics [200]. On this basis, it becomes possible to understand how sensorimotor interactions can mutually alter the respective dynamical landscape of coupled agents. Indeed, when instructed to interact, the differences between participants’ motor signatures shrank (compared to situations where they were not perceptually coupled or where they were instructed to behave individually). While leading another or ignoring the interaction process did not affect much participants motor individuality, having to follow a partner brought participants the furthest away from the behavioral regions they spontaneously visited in solo. Interestingly, improvising jointly (without a designated leader) gave an intermediate picture, where motor dynamics of participants sit in between each other's individual signatures [200]. While this implies less self-differentiation than in the case of followers, this situation brought both participants to move away from their spontaneous repertoire. The meeting of the participants in a place that is now common to them, although it originally belonged to none of their own spontaneous repertoire, results in a creative outcome that is genuinely brought about by the dyad they form. These studies show that using a collaborative creative improvised task allows us to understand how sensorimotor interactions can help an individual to escape from her own determinism, which could potentially lead to novelty, and to meet the other in a joint space that is intrinsic to the relation itself.

In the MG, well synchronized performances also have an aesthetical and affective value as they are correlated with feelings of being more together [198]. In that sense, synchrony is a core factor of success of the improvised interaction, which is not surprising since synchrony is a goal imposed by the MG task instructions. Yet, participants prefer to partially sacrifice the synchrony they are instructed to maintain in order to explore more complex and novel movements, in order to make the interaction more interesting [197, 201]. When modeling this task, it appears that exiting from previously synchronous patterns is a necessary step toward pattern changes, and that noisy fluctuations help shift patterns by increasing the complexity of the set of possible behaviors, thereby fostering the potential discovery of novel patterns [202].

In sum, like in music, members of a dance or movement improvisation group can co-regulate the dynamics of their interaction so as to push them toward dynamical regimes where uncertainty widens the field of opportunities among which novel patterns can be discovered and learnt. Below, we provide an overall discussion of the elements presented above and propose guidelines for future studies of the conditions in which de-synchronization can serve as an effective creative strategy.

## Discussion, future directions and conclusion

In this paper, we have considered creative behavior, and in particular improvisation, from the perspective of dynamical systems. In this view, the behavior of a system is attracted by the stability of specific patterns at the expense of others. For a new pattern to emerge, a previous one has to be disrupted and eventually dismantled. In the context of creativity, such a view anticipates that the disruption of previously established patterns reconfigures the horizon of future possible states and offers an opportunity for DOF of a system to reorganize themselves in a different manner. As such, pattern disruption can foster the exploration of novel patterns of behaviors, thoughts or interactions. Creative shifts have been empirically and phenomenologically demonstrated to be generally preceded by signs of pattern disruption and in particular by transient periods of de-synchronization (e.g., the de-synchronization of the DOF of a motor system, of partners’ movement, or of their intentions). De-synchronization thus appears to be a hallmark of pattern disruption that holds the potential to lead to creative discoveries.

This paper opened up many more questions that were not fully addressed here, including regarding how a creative re-synchronization comes about, and what can teachers, learners or collaborating partners do to bring it about. The topic of creative de- and re-synchronization can be broken down to a number of questions: Which parameters involved in the coordination of a system should be varied in order to produce a creative re-synchronization after disruption? To what extent should the system be de-synchronized for the disruption to foster creative reorganization? What are the socio-affective variables that influence de-synchronization and re-synchronization? Below, we propose preliminary discussions of these issues in order to shape the future directions of the work that the present paper calls for.

### Creative re-synchronization: future directions

When looking at creativity from the angle of *productivity*, for instance by focusing on the originality and successfulness of an improvised performance, and the conditions that allow improvisers to meet those criteria, coordination is a problem that has to be solved. Individuals or groups must “get their acts together” to achieve stable shapes and behavioral sequences. Shifting our attention away from the “productivity” of creative activities allowed us to focus on a key *process* that appears to be necessary for such an outcome to emerge: pattern disruption. From this perspective, the emphasis is more specifically put on creative shifts. This change in focus also shifts the nature of the issue that coordination represents: in order to escape the spontaneous attraction of established patterns, coordination has to be problematized; it is a challenge that needs to be challenged. De-synchronization is a solution to this issue and is integral to the creative activity as it brings forth dynamics that might lead to creative outcome. As such, de-synchronization is integral to the creative process. However, processes that hold the potential to make a creative product emerge often fail or are inconclusive [51], and de-synchronization by itself does not necessarily lead to creative outcomes. In fact, more often than not, after a period of de-synchronization, previously established patterns will re-emerge, reinforcing the challenge of escaping routines and spontaneous tendencies. For instance, the suggestion above regarding varying parameters (such as the tempo) at which movements are executed might annihilate attractors more than it creates new ones, and might therefore encourage re-synchronization of the most established patterns. What, then, are the conditions under which de-synchronization favors creative outcomes? In this section we discuss important issues pertaining to this question.

#### What parameters involved in the coordination of a system should be varied in order to produce a creative disruption?

From a dynamical systems perspective, a crucial issue that has to be addressed is the identification of parameters whose variations can de-synchronize and provoke shifts in the system’s behavior. In group music improvisation, players might increase the complexity of the performed patterns to conjure shifts [139] or play incompatible patterns that break the synchronization with their partner [5]. The introduction of noise in perceptuo-motor loops has also repeatedly been shown to favor creative shifts [34, 72, 202]. When discussing dance learning and improvisation, Miura and colleagues make suggestions regarding the tempo at which movements are performed, because tempo changes can be a source of pattern break-ups and shifts [105]. Indeed, sensorimotor experiments show that changes in tempo at which we coordinate our movement can profoundly affect the landscape of attraction that influences those movements [38]. This might therefore provoke pattern shifts that, with the combination of other variables, could lead to more original patterns.

Moreover, in complex behaviors, finding the parameters whose variation can break up the organization of a system is particularly challenging because it can only be done from the point of view of the dynamical landscape of attraction of the agent(s) to disrupt. Therefore, a knowledge of parameters susceptible to break up patterns is insufficient if we don’t know the dynamical layout it is expected to perturb. In the musical pedagogy of creativity discussed above (see subsection “Improvisation as the problematization of coordination”), the teacher probes the amount of stability of spontaneous patterns and progressively adjusts the amount of disruption as a function of it [5]. Nonetheless, sensing the behavioral regions of attraction of a person and driving their behavior toward alternate paths requires interactional expertise and a repertoire of techniques [5]. Along these lines, rhythmic coordination experiments have been using similar procedures by probing the dynamical landscape of attraction of participants during a particular task [124]. This helped reveal both spontaneous tendencies and unstable patterns which can then be trained during a learning period, the impact of which can then be measured by measuring again the resulting landscape of attraction. In some cases, new attractors appeared, and in others, the whole topology of the landscape was modified [203]. Similar techniques could be used to gauge the spontaneous tendencies of individuals or groups (e.g., rhythmic or melodic patterns in music, sequences of moves in dance). The impact of the variation of different parameters on creative outcomes could be estimated by the extent to which these variations differentiate the spontaneous landscape of attraction.

#### To what extent should the system be de-synchronized for the disruption to foster creative reorganization?

Identifying the right parameters to vary is not enough to ensure a creative disruption. One should also know the magnitude of the applied perturbation. Probing techniques discussed above can be equally used to test the effect of different magnitudes of disruption, and measure how fast it provokes de-synchronization states, how long it makes that state perdure and the extent to which it modifies a dynamical landscape of attraction. In the example of the Kaddouch pedagogy discussed above [5, 174], disruptions often fail at de-synchronizing the learner’s patterns. The teacher has to test different ranges of perturbation to tip the learner to another path. In addition, the constraints placed on the system must not only be strong enough to break its established patterns and make the system less deterministic, but those disrupting constraints must also be sustained for long enough to keep these patterns unstable until another, more creative pattern is enacted. Therefore, in the preceding example, the new constraints imposed by the teacher must be sufficiently strong and should be maintained so as to keep the learner away from her most stable tendencies until an alternative solution has been enacted. On the other hand, there might be an upper limit to the creative efficiency of disruption, above which de-synchronization is beyond repair, or entrains such an amount of uncertainty that it counterproductively leads the participant to get back to the most established patterns, or to the failure of the activity.

#### When should the system be de-synchronized for the disruption to foster creative reorganization?

Cannone reported that “collective sequences” tend to have more or less systematic duration [139] and we mentioned the importance of previous phases of synchronization for building mutual trust and confidence when discussing the Kaddouch pedagogy [5]. Phases of synchronization probably need to last a certain amount of time before a disruption can positively benefit the interaction. In particular, we speculate that disruptions could be detrimental when occurring during moments where players still put in significant effort into fostering their synchronization. However, finding the optimal timing for a ‘good’ disruption is not trivial. In our collective experiences as performers, trainers and facilitators in dance, music and theater improvisation, we often encounter instances where improvisers disrupt too quickly (e.g., not allowing for a stable sequence to be created) or too late (e.g., getting caught in the comfort of the current consensus), while it is really time to move on. Disruptions that come ‘too early’ often bring about confusion, negative interpersonal tensions or even dropping out of the activity. On the other hand, frustration, boredom or lassitude [5] are strong indicators that the interest in the ongoing activity is waning and that a change is called forth. Getting a good sense of the right moment to disrupt an on-going creative process could be one of the qualities that the long term practice and education of improvisation helps foster. Future studies should thus gauge the optimal timing of disruption and in particular, de-synchronization.

#### Re-sync: finding ways to new attractors or finding new ways between known attractors?

While the habitual topology of the landscape of attraction can trap a system in the repetition of its established pattern, it is still in the context of this landscape that reorganization can occur. As such, potentially novel patterns must be enabled by the system’s dynamics and be present, albeit in a latent form, in its repertoire of potential organization. In the gear system experiment above (see subsection “​​From de-synchronization to creative reorganization of agent-environment coordination patterns”), not only is the novel pattern encouraged by the structure of the display, but the behavioral solution is also a sensorimotor pattern that already belonged to the agent’s repertoire. In such a case, what is discovered is that a known pattern has a novel functionality in a new context. In the Kaddouch pedagogy, brand new patterns suddenly emerged. Nonetheless, even when learners abruptly shift to radically novel patterns, they still feature aspects of their initial habits (e.g., tonality, melodic grouping [5]). Yet, these aspects are re-assembled with other features that are uncommon for the player; it is as if both known patterns of DOF and new ones are simultaneously recruited and integrated, making the result sound very novel. In the example with boxers hitting a bag [34, 75], introducing uncertainty encourages the assemblage of novel *combinations* of and *transition* between pre-existing patterns. Because some DOF are coupled with each other to some extent, or even clustered, reorganization after disruption can take those existing links as a scaffold to renew how habitual actions are recombined to form novel sequences [34, 75]. In short, we can face unknown situations creatively by exploiting the dynamics of the network in which DOF are linked with each other, and this allows us to reassemble known patterns into novel sequences or combinations. As such, the framework proposed here is promising for addressing conceptual concerns that have been raised recently [169, 204]. These authors encourage us to reconsider the relation between habits and creativity in a non-dichotomous way and see the habitual aspects of creativity as well as the creative dimension of habits. The dynamical interplay between de-synchronization and re-synchronization processes allows us to understand, within a single explanatory framework, how we can exploit the relations between DOF to not only escape established routines but also creatively renew the functionality of known patterns—in particular by discovering new paths *between* known attractors.

In sum, de-synchronization states are optimal when they let the disrupted situation provide novel affordances that resonate with the system’s latent organization. This entails a number of research questions: what environmental constraints best promote novel reorganization while still resonating with aspects of the system’s intrinsic dynamics? How can we learn to recruit DOF from the environment (including others) to optimally nudge a system toward novel reorganization and overcome the obstacles and lacunae provoked by de-synchronized states? What conditions best encourage a group to re-synchronize through novel sequences of *interpersonal* actions? In the context of improvisation, Canonne [139] points out that certain salient events help motivate pattern change. How, then, can we learn to better attend to, notice and grab the affordances of such events? In dance improvisation, Hansen reminds us of the role and the constraints imposed by cognitive dynamics, in particular the demands that weigh on executive functions during such performances [114]. It is known, for instance, that attentional resources affect the dynamics of interpersonal synchronization [205]. What, then, are the influences of cognitive constraints on the processes of de-synchronization and re-synchronization? How can we best mobilize attentional resources and other executive functions to grab the best opportunities of de-synchronization, or, in a complementary fashion, to foster creative re-synchronization? Those are questions motivated by the proposed framework that future research can address.

#### What are the socio-affective variables that influence de-synchronization and re-synchronization?

Finally, more research should address the socio-affective factors at work or at issue during de- and re-synchronization. In this regard, the existing literature provides seemingly antagonistic results that the account proposed here can help to resolve. Studies on joint action have repeatedly claimed that synchrony, which is often the task goal, is correlated with socio-affective variables such as liking and bonding [206], cooperativeness [207], trust [208], helpfulness [209] and the sense of group efficacy [147]. In the Kaddouch pedagogy, pedagogues also point out the importance of the phases during which they explicitly synchronize with the learner to support her sensorimotor coordination and foster her confidence and trust—it is in such contexts that pedagogues can most safely disrupt the dynamics of interaction [5, 174]. Nonetheless, while synchrony with a partner enhances social cohesion, it does impede the creativity of the interaction with that partner, whether in rhythmic improvisation tasks [210] or in cognitive ones [136]. Indeed, as discussed in this paper, improvisers seek disruption and uncertainty. However states of uncertainty can also induce anxiety and a sense of insecurity that are non-conducive for improvisation. Along these lines, sensorimotor rhythmic experiments have shown that certain interactional roles such as leaders are harder to endorse for those who suffer social anxiety [211]. By decreasing the motivation to leave the comfort zone of established patterns, the anxiety-inducing effects of uncertainty could impede creative exploration.

The framework we propose here suggests an explanatory hypothesis that can reconcile these seemingly antagonistic findings. Rather than contrasting synchrony and asynchrony and taking them as distinct states, the framework proposes to think of synchronization, de-synchronization, and re-synchronization as a dynamical interplay of interdependent processes. Improvisers enjoy playing on the edge between synchrony and asynchrony, and are perhaps more interested in this dialectical process than in the resulting states themselves. From this integrative and dynamic view, it can be argued that the pro-social effects that have been associated with synchrony are not the result of the synchronized states themselves. Rather, they could be a consequence of the feeling that agency is shared through mutual interactions during which partners navigate synchronization, de-synchronization, and re-synchronization. Consequently, realizing that we can overcome transient periods of uncertainty together should reinforce mutual trust. In fact, from sensorimotor [212] and improvisational experiments [201] to research on mother-infant interactions [213], several recent studies show that interpersonal synchrony is often sacrificed in the short-term to foster coordination in the long-term. Relatedly, in the MG, it has been shown that secure attachment allowed partners to improvise with more complexity and less synchronicity [214]. Group improvisation thus both relies on and fosters prosocial feelings such as mutual trust, and the confidence that sharing agency, rather than controlling it individually, can help reach joyful states where creativity flows through the dialectic between de-synchronization and re-synchronization. The fact that improvisation specifically relies on and exploits the interdependence between these processes further makes it a powerful clinical intervention tool [114, 215].

The central claim of this paper regarding the importance of disruption and more particularly de-synchronization for creative processes seems to go against a common preconception that considers these phenomena as an issue to overcome [158]. Clearly, not all forms of disruption or de-synchronization are necessarily positive. Disruptions can lead to the failure or degradation of creative processes. For disruption to foster creativity, it should put agents in situations they feel like they can recover from. Disruptions should thus motivate explorational behavior, but not make the system’s activity collapse entirely beyond possible repairs, or discourage the agent from taking part in the creative activity. Along these lines, the “honing theory” proposes that the amount of uncertainty within one’s own current cognitive organization has a phenomenological counterpart (“felt entropy”): above a certain threshold, felt entropy provokes an arousal that calls for attention, and motivates exploratory behaviors and pattern reorganization [52]. Such a threshold is likely to be agent- or group-dependent, as creativity has often been associated with personality traits such as risk-taking and tolerance of ambiguity [216]. As we discussed earlier, the creative foraging paradigm provides evidence for a correlation between the length of time a player spent exploiting a pattern and the length of the following exploration of new ones [45]. This result captures the inherent interpersonal differences in creative foraging that might indicate different levels of risk-taking tolerance: each person chooses when to disrupt exploitation differently. The correlation of exploitation and exploration times might also suggest that players optimize their disruption timing as a function of how quickly they expect subsequent explorations to bear fruits. In other words, risk taking (disrupting the exploitation of a known territory) is modulated by the prospects of the following exploration. Beyond creative foraging, the idea of optimization of foraging behavior was widely discussed in animal [217] and human [218, 219] foraging studies. Future research can address the relation between the honing theory construal [52] and the optimization principle suggested here.

Other elements could also modulate the balance between exploration and exploitation, such as the level of stress that the environment imposes on the agent or the group. For example, in improvisation, the presence of an audience might increase performers’ level of anxiety, inciting them to avoid uncertainty and stay within the comfort zones of their established patterns. In such a situation, partners treat their collective performance as a *coordination problem* (trying to maximize their coordination) instead of treating their collective performance as a *problematization of coordination* (trying to disrupt their habitual, established patterns of interaction).

In sum, for creative re-synchronization to take place, the disruption caused by de-synchronization must not entirely impeach the sensorimotor agency of the individuals or the group, and does not need to prescribe specific patterns. Rather, constraints and disruption must help an agent or a group navigate its own potentials by unveiling a novel space of opportunities that can be grabbed on to, and that are related sufficiently enough to stable aspects of the system’s intrinsic dynamics so that novel patterns can be enacted with a certain level of stability.

## Conclusion

In this paper, we present a new perspective on creative processes. Through the lens of dynamical systems, and by linking experimentally observed behavior to the pragmatic experience of practitioners, we propose that disruption of established patterns plays a key role in creative processes and that de-synchronization is a hallmark of such disruptive moments. We further suggest that de-synchronization, by raising uncertainty, provides opportunities of re-synchronization and reorganization into novel functional patterns to discover, explore, and ultimately, learn. The interplay between de-synchronization and (re-)synchronization, which naturally results from the non-linear coupling between DOF, can thus account for the emergence of novelty (or the “creation of information”—[65]) without postulating processes or modules that are dedicated to creativity per se. Instead, this interplay potentially accounts for creative dynamics through the interaction between sub-elements, which constitutes a big ambition for creativity research [220].

Importantly, the proposed framework spans individual and group levels of organization, which are usually treated separately in the literature, but can now be looked at from a similar angle and investigated with similar tools and concepts. The proposed perspective thus offers a pragmatic frame for a strategy of teaching methods that can be enacted to help people create together, and to help them learn through the paths of creative processes. Such an issue is not only important for educational contexts, but also for scientific research itself, a creative activity that is more and more collaborative [221], but whose process tends to get less disruptive over time [222].

Finally, the present proposition resonates with recent and more general accounts such as “irruption theory” [223], which states that the more general ability to exert agency involves a capacity to provoke transient bursts of unpredictability in one’s own neurophysiological processes. In other words, agency could itself be a form of disruption. Therefore, the ability to get creative would, in that sense, be in direct continuity with the ability to exert agency: the capacity of establishing, losing and re-establishing meaningful coupling with the world [224]. As such, studying creativity and improvisational practices in particular might enlight more general aspects of the human condition.

## Data Availability

Not applicable.

## Abbreviations

CI: Contact improvisation
DOF: Degrees of freedom
DS: Disrupted state
FS: Final state
IS: Initial state
MG: Mirror game
